# Draft genome sequence of Japanese wood mouse, *Apodemus speciosus*

**DOI:** 10.1016/j.dib.2017.10.063

**Published:** 2017-10-31

**Authors:** Masatoshi Matsunami, Daiji Endo, Naruya Saitou, Hitoshi Suzuki, Manabu Onuma

**Affiliations:** a Laboratory of Ecology and Genetics, Graduate School of Environmental Science, Hokkaido University, Kita-ku, Sapporo 060-0810, Japan; b School of Veterinary Medicine, Rakuno Gakuen University, Ebetsu 069-8501, Japan; c Department of Genetics, School of Life Science, The Graduate University for Advanced Studies (SOKENDAI), Mishima, 411-8540, Japan; d Ecological Risk Assessment and Control Section, Center for Environmental Biology and Ecosystem Studies, National Institute for Environmental Studies, 16-2, Onogawa, Tsukuba, Ibaraki 305-8506, Japan; e Graduate School of Medicine, University of the Ryukyus, Nishihara-cho 903-0215, Japan

**Keywords:** Rodent, Mouse, *Apodemus speciosus*, Phylogeography, Mammal, Evolution

## Abstract

The wood mouse (genus *Apodemus*) is one of the most common rodents in broad-leaf forests in the temperate zone of the Palaearctic region. Molecular studies of wood mice have critically enhanced the understanding of their evolution and ancestral biogeographic events. However, their molecular data are currently only limited to partial mitochondrial sequences and a few genes. Therefore, we sequenced the wood mouse genome to facilitate the acquisition of useful resources for inferring their molecular evolution. We sampled a wild wood mouse at Tsukuba, Japan, and sequenced its whole-genome using the Illumina Hiseq. 2000. To reduce the risk of non-randomness, three paired-end libraries (insert sizes: 150, 300, and 500 bp) and, two mate-pair reads (insert sizes: 8 and 20 kbp) were constructed. In total, we generated approximately 210 Gbp data. From these sequences, we reconstructed 336,124 scaffolds. These data will enhance our understanding of the evolution and ecological factors that affect their genetic constitution. The genome scaffolds generated are available in the National Center Biotechnology Information (NCBI) BioProject with accession number PRJDB5914.

**Specifications Table**

**Table:** 

**Subject area**	*biology*
**More specific subject area**	*Genomics*
**Type of data**	*Genome sequences*
**How data was acquired**	*High throughput DNA sequencing using Hiseq. 2000*
**Data format**	*FASTA format*
**Experimental factors**	*Total DNA extraction from liver samples*
**Experimental features**	*Freshly collected healthy liver was used for total DNA extraction*
**Data source location**	*Tsukuba, Ibaraki, Japan*
**Data accessibility**	*The genome scaffolds generated are available in the National Center Biotechnology Information (NCBI) BioProject with accession number*PRJDB5914.
**Related research article**	*Not applicable*

**Value of the Data**

• The Japanese wood mouse could be a model mammal for investigating the natural histories of organisms inhabiting the Japanese Archipelago.
• We sequenced the Japanese wood mouse genome to facilitate the acquisition of useful resources for inferring the molecular evolution of this species.
• This data allows other researchers focusing on this species to start genome-wide analysis.

## 1 Data

The genus *Apodemus,* which is a member of the subfamily Murinae, is one of the most species rich taxa of the murine rodents, next to *Rattus* and *Mus*, which are the first and second speciose genera, respectively [1]. Species belonging to *Apodemus*, known as field or wood mice, are distributed in the temperate region of the Eurasian continent including the Japanese Archipelago. According to previous molecular phylogenetic analyses, *Apodemus* diversified in Asia and Europe, around the early Pliocene and early Pleistocene eras, respectively [2]. Deciphering the genome sequences of this genus would facilitate our understanding of the evolutionary trait of the group adapted to the temperate environment, with the aid of those of *Rattus* and *Mus*. The genome of a representative species of *Apodemus* from Europe, *Apodemus sylvaticus*, was sequenced by a research group at the Liverpool University according to the JGI GOLD database (https://gold.jgi.doe.gov/; project ID = Gp0143296), but its project status is "permanent draft." In Asian species, no genome sequence data are currently available.

The large Japanese wood mouse, *Apodemus speciosus*, is widely distributed in Japan from Hokkaido to Kyushu, including the remote peripheral islands. This species inhabits forests and grasslands, eats insects and acorns, and shows seasonal breeding in the wild. A recent phylogeographic analysis revealed that this species experienced a rapid population growth in response to the effect of the Quaternary ice ages [3]. Thus, the species could be a model mammal for investigating the natural histories of organisms inhabiting the Japanese Archipelago [4]. We sequenced the Japanese wood mouse genome to facilitate the acquisition of useful resources for inferring the molecular evolution of this species.

The genome was assembled using the ALLPATHS-LG program using two-step processes of assembling and scaffolding. The paired-end reads were first assembled into contigs, which were then joined to form concatenated scaffolds by using mate-pair reads. We reconstructed 647,983 contigs (N_50_ is 3.5 kbp). These contigs were joined in 336,124 scaffolds (Table 2), and the N_50_ value of the scaffolds was 47 kbp. The wood mouse genome sequenced is a potentially useful resource for the future molecular evolutionary analysis of rodents.

## 2 Experimental design, materials, and methods

### 2.1 Sample preparation and sequencing

We sampled the *A. speciosus* at Tsukuba, Japan (NIES ID; IB14-021, male). All rodent experiments were conducted in accordance with the guidelines for using wild mammals of the Mammal Society of Japan [5] and the rules of the National Institute for Environmental Studies for analysis and experimentation with environmental samples contaminated with radioactive materials. Genomic DNA was extracted from the liver using proteinase K and phenol:chloroform:isoamyl alcohol [6]. The library preparations and sequencings were conducted by the Eurofin Sequencing Service (Eurofin MWG Operon, Germany). Paired-end sequencing of each 100-bp end of the DNA fragments was performed using the Hiseq. 2000 sequencing system (Illumina, San Diego, CA, USA). We sequenced libraries showing five different insert sizes: 150 bp, 300 bp, 500 bp, 8 kbp, and 20 kbp.

### 2.2 De novo genome assembly

We sequenced three paired-end libraries (insert sizes: 150, 300, and 500 bp) and two mate-pair reads (insert sizes: 8, and 20 kbp). In total, we generated approximately 210 Gbp data. We obtained 362107447, 336419570, 369663105, 22370797, and 12377116 reads for each library showing 150-bp, 300-bp, 500-bp, 8-kbp, and 20-kbp insert sizes, respectively (Table 1). The sequenced reads were used for de novo genome assembly using the ALLPATHS-LG program ver. 51927 under default parameter settings [7].

**Table 1:** Data amount.

Insert size (bp)	No. of read pairs	No. of reads	Read length (bp)	Total data (GB)
150	362,107,447	724,214,894	100	72.4
300	336,419,570	892,839,140	100	89.3
500	369,663,105	739,326,210	100	73.9
8000	22,370,797	44,741,594	100	4.5
20,000	12,377,116	24,754,232	100	2.5

**Table 2:** General features of the *Apodemus speciosus* genome.

Number of contigs	647,983
Number of contigs per megabase (Mb)	189
Number of scaffolds	336,124
Total contig length	2,009,551,775
Total scaffold length, with gaps	3,428,181,270
N_50_ contig size (kb)	3.5
N_50_ scaffold size (kb)	25
N_50_ scaffold size (kb), with gaps	47
Number of scaffolds per megabase (Mb)	98.05
Median size of gaps in scaffolds	2846
Median deviation of gaps in scaffolds	852

## References

[1] Schenk J.J., Rowe K.C., Steppan S.J. (2013). Ecological opportunity and incumbency in the diversification of repeated continental colonizations by muroid rodents. Syst. Biol..

[2] Serizawa K., Suzuki H., Tsuchiya K. (2000). A phylogenetic view on species radiation in Apodemus inferred from variation of nuclear and mitochondrial genes. Biochem. Genet..

[3] Tomozawa M., Suzuki H. (2008). A trend of central versus peripheral structuring in mitochondrial and nuclear gene sequences of the Japanese wood mouse, Apodemus speciosus. Zool. Sci..

[4] Suzuki Y., Tomozawa M., Koizumi Y., Tsuchiya K., Suzuki H. (2015). Estimating the molecular evolutionary rates of mitochondrial genes referring to Quaternary Ice Age events with inferred population expansions and dispersals in Japanese Apodemus. BMC Evol. Biol..

[5] Committee of Reviewing Taxon Names and Specimen Collections (2009). Guidelines for using wild mammals. Mamm. Sci..

[6] Sambrook J., Russel D.W. (2001). Preparation of Genomic DNA from Mouse Tails and other Small Samples, in Molecular Cloning: a Laboratory Manual.

[7] Gnerre S., Maccallum I., Przybylski D., Ribeiro F.J., Burton J.N., Walker B.J., Sharpe T., Hall G., Shea T.P., Sykes S., Berlin A.M., Aird D., Costello M., Daza R., Williams L., Nicol R., Gnirke A., Nusbaum C., Lander E.S., Jaffe D.B. (2011). High-quality draft assemblies of mammalian genomes from massively parallel sequence data. Proc. Natl. Acad. Sci. USA.

