# Supplementary material for: Draft genome sequence of Japanese wood mouse, Apodemus speciosus
Source: Data Brief. 2017 Oct 31;16:43–6. doi: 10.1016/j.dib.2017.10.063 (PMC5848110; doi:10.1016/j.dib.2017.10.063)
Supplement: Supplementary file 1 — Supplementary material [file mmc1.docx]

Hao-Ran Wang

Editor-in-Chief

Data in Brief

Dear Dr. Hao-Ran Wang:

This manuscript has not been published or presented elsewhere in part or in entirety and is not under consideration by another journal. We have read and understood your journal’s policies, and we believe that neither the manuscript nor the study violates any of these. There are no conflicts of interest to declare.

Thank you in advance for your kind consideration of this paper.

Sincerely yours,

Manabu ONUMA

Ecological Risk Assessment and Control Section,

Center for Environmental Biology and Ecosystem,

National Institute for Environmental Studies,

16-2, Onogawa, Tsukuba, Ibaraki, 305-8506, Japan.

TEL: 029-850-2498 Fax: 029-850-2673,

E-mail: [monuma@nies.go.jp](mailto:monuma@nies.go.jp)
